# Integrating Ontogenetic Shift, Growth and Mortality to Determine a Species' Ecological Role from Isotopic Signatures

**DOI:** 10.1371/journal.pone.0125059

**Published:** 2015-05-21

**Authors:** Nelson F. Fontoura, Lúcia R. Rodrigues, Cibele B. Batista, Tanilene S. P. Persch, Mariola E. Janowicz

**Affiliations:** 1 Faculdade de Biociências; Pontifícia Universidade Católica do Rio Grande do Sul, Porto Alegre, Rio Grande do Sul, Brazil; 2 Instituto de Pesquisas Hidráulicas, Universidade Federal do Rio Grande do Sul, Porto Alegre, Rio Grande do Sul, Brazil; 3 Department of Biology and Environmental Sciences, Concordia University College of Alberta, Edmonton, Alberta, Canada; University of Otago, NEW ZEALAND

## Abstract

Understanding species linkages and energy transfer is a basic goal underlying any attempt at ecosystem analysis. Although the first food-web studies were based on gut contents of captured specimens, the assessment of stable isotopes, mainly *δ^13^C* and *δ^15^N*, has become a standard methodology for wide-range analyses in the last 30 years. Stable isotopes provide information on the trophic level of species, food-web length, and origin of organic matter ingested by consumers. In this study, we analyzed the ontogenetic variability of *δ^13^C* and *δ^15^N* obtained from samples of three Neotropical fish species: silver sardine (*Lycengraulis grossidens*, *n*=46), white lambari (*Cyanocharax alburnus*, *n*= 26), and the red-tail lambari (*Astyanax fasciatus*, *n*=23) in Pinguela Lagoon, southern Brazil. We developed a new metric, called the Weighted Isotopic Signature (*φ^ 15^N* or *φ^ 13^C*, ‰), that incorporates ontogenetic variability, body growth, and natural mortality into a single number.

## Introduction

Ecosystems are complex by nature, and interactions and patterns can be obscured by many processes that occur simultaneously at different spatial and temporal scales [1]. In this regard, the use of stable isotopes is a valuable tool to record and integrate ecological patterns and processes [2]. Heavy isotopes of carbon and nitrogen (^13^C and ^15^N) are scarcer in nature than the lighter ones (^12^C and ^14^N). The ratio of the heavier to lighter forms, expressed as *δ* ^*13*^ *C* and *δ* ^*15*^ *N*, can be very informative of both the main energy source and the trophic position of a species [3–8]. The ratio of carbon isotopes can vary according to the photosynthetic metabolism, and different producers can show distinct *δ* ^*13*^ *C* signatures [4]. Because the carbon-isotope ratio changes little along a trophic chain, typically increasing < 1‰ in each trophic level [3–9], it can be used to trace the primary food sources of a single species or for the entire food web of an ecosystem. On the other hand, nitrogen isotope ratios normally increase from 2.5‰ to 3.5‰ between successive trophic levels, and are also used to establish the trophic position of species [3–10].

Nevertheless, using stable isotopes to depict a food web is not an easy task, because the isotope signatures of species change as animals grow from the larval or juvenile to the adult stage [11], reflecting an ontogenetic shift in both the primary energy source and the trophic position. But if we consider that in nature, young animals are much more abundant whereas large individuals are relatively less numerous, how can we characterize a species by using a single number? How can we summarize a species' trophic role in a food web?

In the present study, we analyzed how ontogenetic variability within a species could be managed, taking into account both animal growth and mortality. We developed a new metric, called the Weighted Isotopic Signature (*φ* ^*15*^ *N* or *φ* ^*13*^ *C*, ‰), which incorporates all the ontogenetic variability, animal body growth, and natural mortality into a single number. If a species must be represented as a single number expressing an isotopic signature, our proposal aims to reduce the sources of bias.

## Methods

### Data source

We collected three fish species: *Lycengraulis grossidens* (Spix & Agassiz, 1829) (silver sardine), *Cyanocharax alburnus* (Hensel, 1870) (white lambari) and *Astyanax fasciatus* (Cuvier, 1819) (red-tail lambari) in Pinguela Lagoon, southern Brazil (29°49'S, 050°10'W; November and December 2009, and March and April 2010) by using a beach seine net (50 m long, mesh size 5 mm; collecting permit issued by the Federal Environmental Agency, IBAMA, # 10200–2). These species are common in the area and are not endangered or vulnerable. The species were selected because of their different ontogenetic shifts in feeding. The fish were euthanized by immersion in a solution of 285 μg.L^-1^ eugenol, and all procedures were approved by the institutional Committee for Ethical Animal Use (CEUA; http://www.pucrs.br/portal/?p=pesquisa/ceua). Specimens from the same locality are deposited in the Museu de Ciências e Tecnologia, PUCRS: *L*. *grossidens*, MCP 13879; *C*. *alburnus*, MCP 45673; and *A*. *fasciatus*, MCP-45672.

Stable isotope ratios of carbon and nitrogen (*δ* ^*13*^ *C*, *δ* ^*15*^ *N*) of measured specimens of *C*. *alburnus* (*n* = 26), *A*. *fasciatus* (*n* = 23) and *L*. *grossidens* (*n* = 46) were quantified from samples of dried muscle ground to a fine powder (weighed to 10^–6^ g) and placed in Ultra Pure tin capsules (Costech Analytical Technologies, Valencia, CA, USA). Samples were sent to the UC Davis Stable Isotope Facility, Department of Plant Sciences at the University of California, and analyzed by means of a PDZ Europa ANCA–GSL elemental analyzer interfaced to a PDZ Europa 20–20 isotope ratio mass spectrometer (Sercon Ltd, Cheshire, UK). A preliminary isotope ratio was measured relative to reference gases of the included laboratory standards. The long-term standard deviation was 0.2‰ for ^13^C and 0.3‰ for ^15^N. The final delta values are expressed relative to the international standards V-PDB (Vienna PeeDee Belemnite) and Air for carbon and nitrogen, respectively.

The potential ontogenetic shift of isotope values in relation to fish size was tested using linear and logarithmic regressions (SPSS ver. 17.5). Complex patterns resulting from ontogenetic shifts in isotopic signatures were described through polyphasic models [12]. Essentially, a polyphasic function is a series of regular simple functions, each describing a specific response for a period of the animal’s life cycle, all merged together by switch (logistic) equations to turn each specific life cycle stanza on or off: $S_{w}={\left(1+e^{T_{x}\left(L-SCL\right)}\right)}^{-1}$; where *S*_*w*_ is the interruption factor (ranging from one to zero), *T*_*x*_ is the rate of change, *L* is the standard length (mm), and *SCL* is the mean length (cm) at which individuals switch from one stanza to the next. Once the life stanzas were identified, polyphasic functions were adjusted by using a nonlinear regression routine of SPSS (Levenberg-Marquardt algorithm).

### Integrating ontogenetic shift, growth and mortality

We propose a model aiming to merge information on growth, mortality and diet shift, measured throughout a species' life cycle. To simplify the model, we considered a population that shows a survival response with a constant mortality rate [13], although more-complex approaches could be applied [14]:

$$l_{t}=\;e^{-Mt}$$ (1)

Where *l*_*t*_ is the probability that an individual will survive to age *t* (years); *M* is the instantaneous mortality rate; and *t* is the age (years).

Although growth could follow complex seasonal patterns [15], we will simplify it by using a regular von Bertalanffy growth model:

$$L_{t}=L_{\infty }\left(1-e^{-K\left(t-t_{0}\right)}\right)$$ (2)

Where *L*_*t*_ is the mean length (cm) at age *t* (years); *L*_*∞*_ is the asymptotic length (cm); *K* is the instantaneous growth rate; *t* is the age (years); and *t*_*o*_ is a time correction factor related to size at birth (years).

The weight/length relationship is estimated as a power function [16]:

$$W=aL^{b}$$ (3)

Where *W* is the total weight (g) at total length *L* (cm); *a* is the proportionality coefficient; and *b* is the allometric coefficient.

The weight at age *t* (*W*_*t*_) was obtained by merging functions 2 and 3:

$$W_{t}=a(L_{\infty }{\left(1-e^{-K\left(t-t_{0}\right)}\right)}^{b}$$ (4)

Where all parameters have been previously defined.

The expected biomass per recruit (*B*_*t*_) at age *t* is the product of Eqs 1 and 4:

$$B_{t}=\;l_{t}W_{t}$$ (5)

Population parameters (*M*, *L∞*, *K*, *a*, *b*) were obtained in the FishBase data source [17] and previously published literature [18, 19].

The isotopic signature may follow complex patterns (linear, logarithmic or polyphasic) during the ontogenetic development of a species, and is described as a universal function:

$$\delta_{L}^{15}N\;or\;\delta_{L}^{13}C=f\left(L_{t}\right)$$ (6)

Where *δ*_*L*_^*15*^ *N* or *δ*_*L*_^*13*^ *C* (‰) is the isotopic signature at length *L*_*t*_ (cm). Once the $\delta_{L}^{15}N\;\text{or}\;\delta_{L}^{13}C$ to length function is obtained, it can be easily converted to an age function by using the von Bertalanffy growth curve (Eq 2):

$$\delta_{t}^{15}N\;\text{or}\;\delta_{t}^{13}C=f\left(L_{\infty }\left(1-e^{-K\left(t-t_{0}\right)}\right)\right)$$ (7)

The Weighted Isotopic Signature (*φ* ^*15*^ *N* or *φ* ^*13*^ *C*, ‰) of a species could be interpreted as a composite average of δ^*15*^ *N* or δ^*13*^ *C*, taking into account its age-specific isotopic signature and its relative abundance (as biomass), and is defined as:

$$\text{Species composite impact per recruit}:\;\left(\delta_{t}^{15}N\;\text{or}\;\delta_{t}^{13}C\right)\;B_{t}$$ (8)

$$\text{Total biomass per recruit}:\;\int \;B_{t}\;dt$$ (9)

$$\varphi^{15}N\;\text{or}\;\varphi^{13}C=\frac{\int [(\delta_{t}^{\;15}N\;\text{or}\;\delta_{t}^{\;13}C)\;B_{t}]\;dt}{\int \;B_{t}\;dt}$$ (10)

The numerator ($\int {[(\delta }_{t}^{\;15}N\;\text{or}\;\delta_{t}^{\;13}C)\;B_{t}]\;dt$) is the integral solution of the product of Eqs 7 and 5, considering the impact of each age on the species' isotopic signature. The denominator expresses the integral solution for the biomass function, representing the total biomass per recruit for the species.

Solutions for the proposed integrals must be solved on a case-by-case basis, depending on the mathematical function selected to describe the ontogenetic variation of the isotopic signature, by using mathematical software (such as Maple V). An approximate and simpler solution would be to divide the size range of the species into *n* discrete classes (the more the better), considering the isotopic signature with the expected biomass for each size class, as follows:

$$\varphi^{15}N\;\text{or}\;\varphi^{13}C=\frac{\sum_{i=1}^{n}(\delta_{t}^{\;15}N\;\text{or}\;\delta_{t}^{\;13}C)\;B_{t}}{\sum_{i=1}^{n}B_{t}}$$ (11)

## Results

### Ontogenetic variability

**Fig 1:**
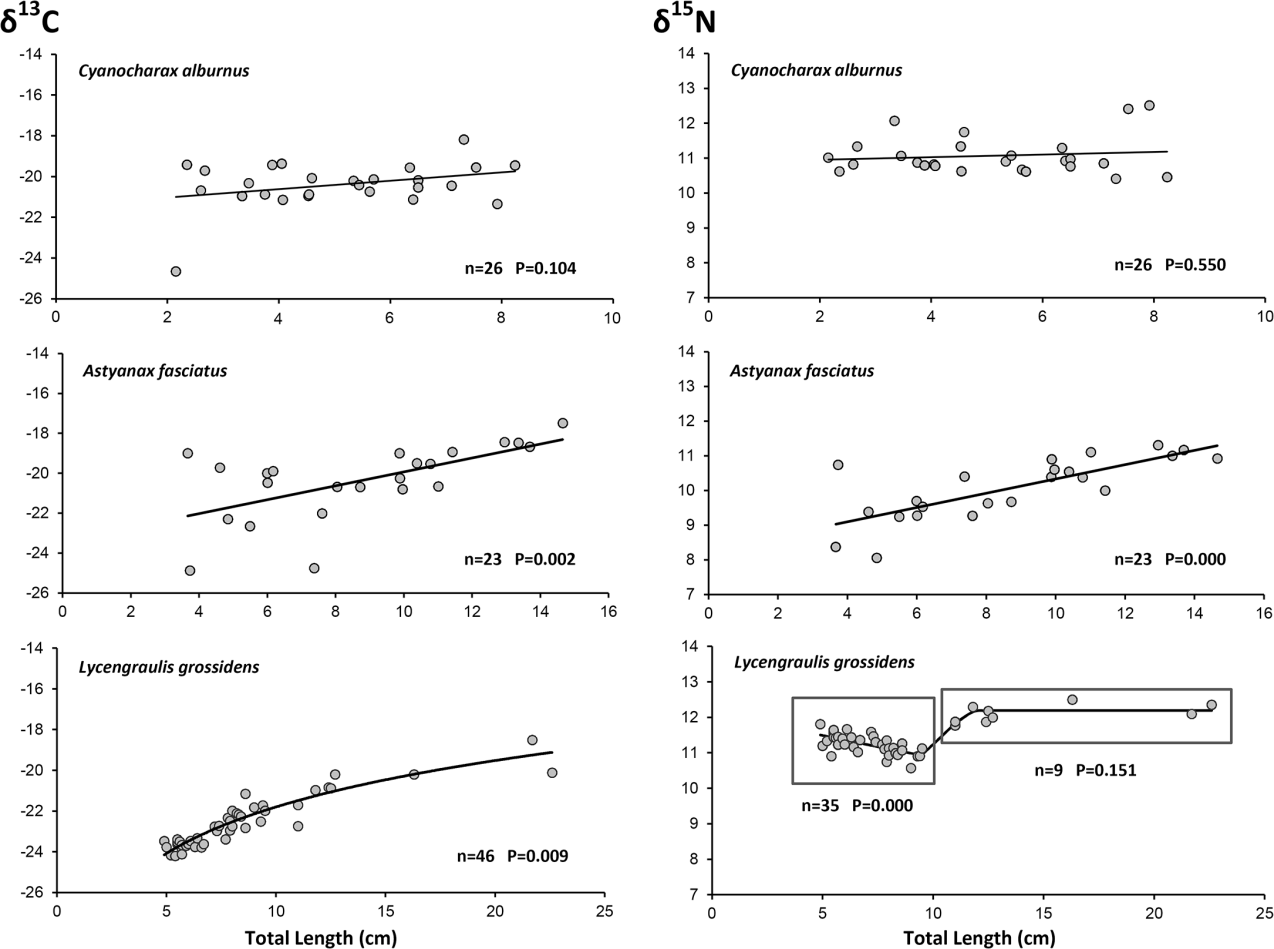
Ontogenetic variability of isotope signatures of carbon (*δ* ^*13*^ *C*) and nitrogen (*δ* ^*15*^ *N*) for *Cyanocharax alburnus*, *Lycengraulis grossidens* and *Astyanax fasciatus* in Pinguela Lagoon, southern Brazil.

The ontogenetic shift in isotope values (Fig 1) was not significant for *C*. *alburnus* (linear regression; P>0.05), but was highly significant for *A*. *fasciatus*

($\delta_{L}^{13}C=0.348L_{t}-23.4$, P = 0.002; $\delta_{L}^{15}N=0.205L_{t}+8.28$, P≤0.001) and *L*. *grossidens* ($\delta_{L}^{13}C=3.29\text{ln}(L_{t})-29.4$, P = 0.009). *L*. *grossidens* also showed a complex pattern for nitrogen isotope values (polyphasic regression), decreasing linearly for individuals under 10 cm (P<0.001), then increasing abruptly and subsequently remaining steady (constant value, no slope significance, P = 0.151). The mathematical solution for this complex pattern (Eq 12) includes a simple linear function for small animals (Stanza 1), a constant value for larger ones (Stanza 2), and a logistic function acting as a switch (*S*_*w*_) that modulates the transition from stanza one to stanza two:

$$\text{Switch Function}:\;S_{w}={\left(1+e^{3.20\left(L_{t}-10.7\right)}\right)}^{-1}$$

$$\text{Stanza}\;1:\;\delta_{L}^{15}N\;\left(1\right)=\left(12.1-0.127L_{t}\right)\left(S_{w}\right)$$

$$\text{Stanza}\;2:\;\delta_{L}^{15}N\;\left(2\right)=12.2\;\left(1-S_{w}\right)$$

$$\delta_{L}^{15}N=\;\delta_{L}^{15}N\;\left(1\right)+\;\delta_{L}^{15}N\;\left(2\right)$$ (12)

### Integrating ontogenetic shift, growth and mortality

As there is no isotopic ontogenetic shift for *C*. *alburnus*, this species does not require estimation of a Weighted Isotopic Signature, and a simple average of isotopic signature values will be enough to represent the species' ecological role. On the other hand, as ontogenetic patterns were identified for *L*. *grossidens* and *A*. *fasciatus*, information concerning growth and mortality parameters is needed (Table 1). To make the proposal easier to follow, a step-by-step example for *A*. *fasciatus* is presented, using the *δ* ^*15*^ *N* ontogenetic shift.

**Table 1:** Growth, mortality and isotopic parameters (‰) for *Astyanax fasciatus* and *Lycengraulis grossidens* in Pinguela Lagoon, southern Brazil.

	*L*. *grossidens*	*A*. *fasciatus*
Asymptotic length (L_∞_; cm)	24.3	13.0
Instantaneous growth rate (K; year^-1)^	0.27	1.16
Proportionality constant (a)	0.0033	0.0099
Allometric constant (b)	3.29	3.06
Natural mortality (M; year^-1^)	0.59	1.78
$\int \delta_{L}^{\;15}N\;B_{t}\;dt$	179.16	20.13
$\int \delta_{L}^{\;13}C\;B_{t}\;dt$	-303.57	-39.82
∫ *B*_*t*_ *dt*	14.87	1.97
Average δ^*15*^ *N* values (‰)	11.41	10.07
*φ* ^*15*^ *N* (‰)	12.05	10.20
Average *δ* ^*13*^ *C* values (‰)	-22.59	-20.38
*φ* ^*13*^ *C* (‰)	-20.42	-20.17

**Fig 2:**
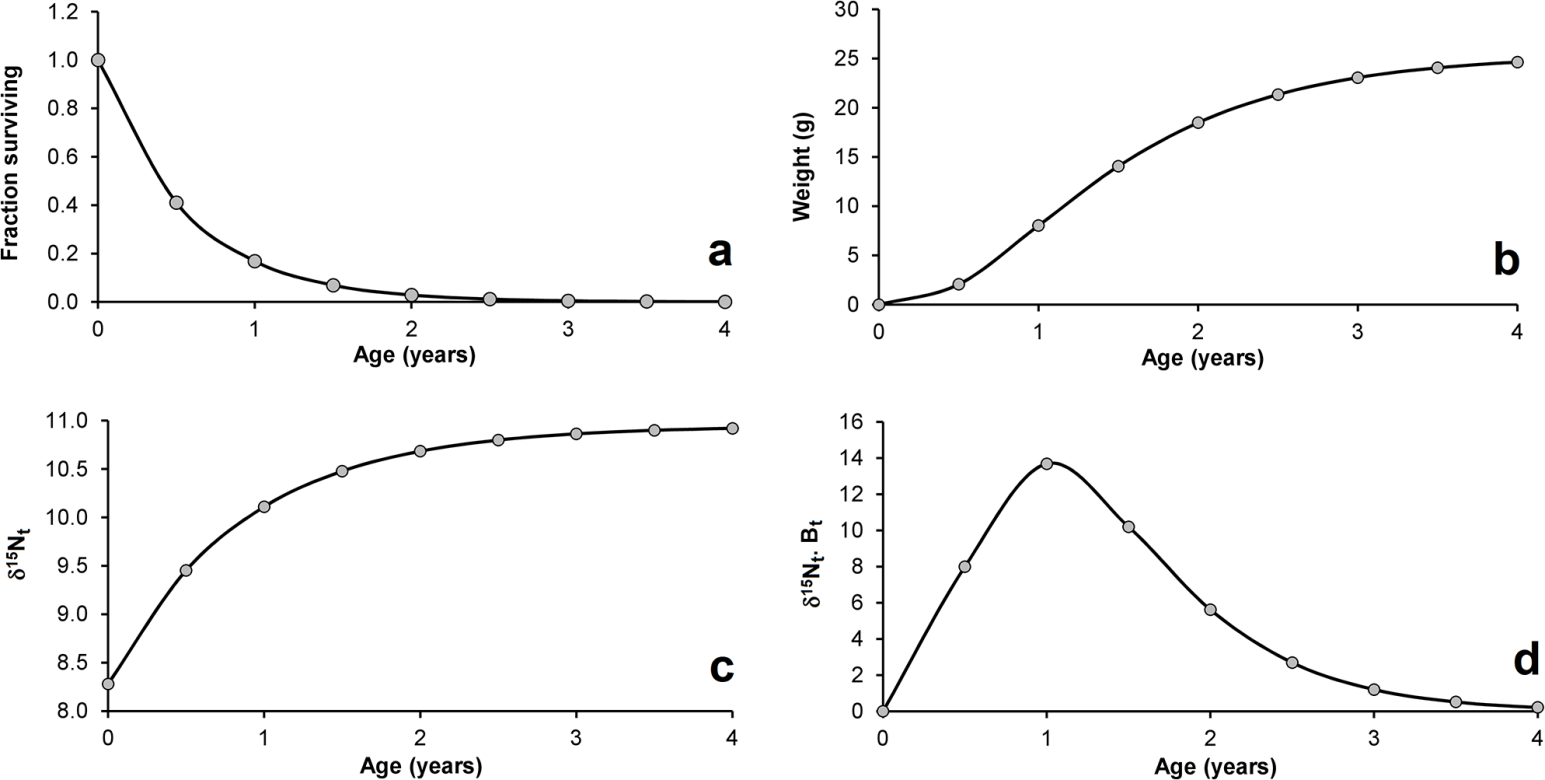
*Astyanax fasciatus* in Pinguela Lagoon, southern Brazil: (a) survival curve; (b) weight growth curve; (c) *δ* ^*15*^ *N*-to-age response; (d) weighted impact of *δ* ^*15*^ *N* according to age.

Starting from birth, the probability of survival decreases exponentially, with almost no individual reaching the age of four (Eq 1; M = 1.78; Fig 2A). With an asymptotic length (*L∞*) of 13 cm, growth constant (*K*) of 1.16, proportionality constant (*a*) of 0.0099, and allometric constant (*b*) of 3.06, the species grows asymptotically to ± 25 g according to the von Bertalanffy growth formula (Eq 4; Fig 2B). By converting the *δ* ^*15*^ *N*-to-length response ($\delta_{L}^{15}N$; Fig 1) into the *δ* ^*15*^ *N*-to-age response ($\delta_{t}^{15}N$; Eq 7; Fig 2C), a pattern very similar to a von Bertalanffy growth curve for length can be achieved, expressing not the length-to-age, but the expected *δ* ^*15*^ *N*-to-age relationship:

$$\text{Nitrogen signature from length}:\;\delta_{L}^{15}N=0.205L_{t}+8.28$$

(already described)

$$\text{Length to age}:\;L_{t}=13.0(1-e^{-1.16t})$$

(Eq 2; Table 1 values)

$$\text{Nitrogen signature to age}:\;\delta_{t}^{15}N=0.205[13.0(1-e^{-1.16t})]+8.28$$ (13)

By multiplying the probability of survival (Fig 2A) by the growth curve for weight (Fig 2B), a curve of biomass per recruit can be obtained (Eq 5, not shown); and by multiplying the nitrogen-to-age function (Eq 13; Fig 2C) by the biomass per recruit, we can estimate the weighted impact of individuals at any size in terms of the nitrogen signature (Eq 8; Fig 2D). The integral of this function divided by the integral of the biomass per recruit represents the Weighted Isotopic Signature (*φ*; Eq 10).

The biomass per recruit (∫ B_t_ dt) is 14.87 g and 1.97 g for *L*. *grossidens* and *A*. *fasciatus* respectively (Table 1). Solutions for the Weighted Isotopic Signature (*φ* ^*15*^ *N* or *φ* ^*13*^ *C*, ‰) did not deviate from the average isotopic signature including all fish sampled. For *A*. *fasciatus*, the average *δ* ^*15*^ *N* was 10.07‰ whereas the Weighted Isotopic Signature (*φ* ^*15*^ *N)* was estimated at 10.20‰ (Δ = 0.13). Similarly, for carbon signatures, the average *δ* ^*13*^ *C* was -20.38‰ whereas *φ* ^13^C was estimated at -20.17‰ (Δ = 0.21). The differences were larger for *L*. *grossidens*, with *φ* ^*15*^ *N* slightly exceeding the *δ* ^*15*^ *N* average by about 0.6‰. More-significant differences were observed in the carbon values, with a 2‰ difference between the *φ* ^*13*^ *C* and *δ* ^*13*^ *C* averages.

**Fig 3:**
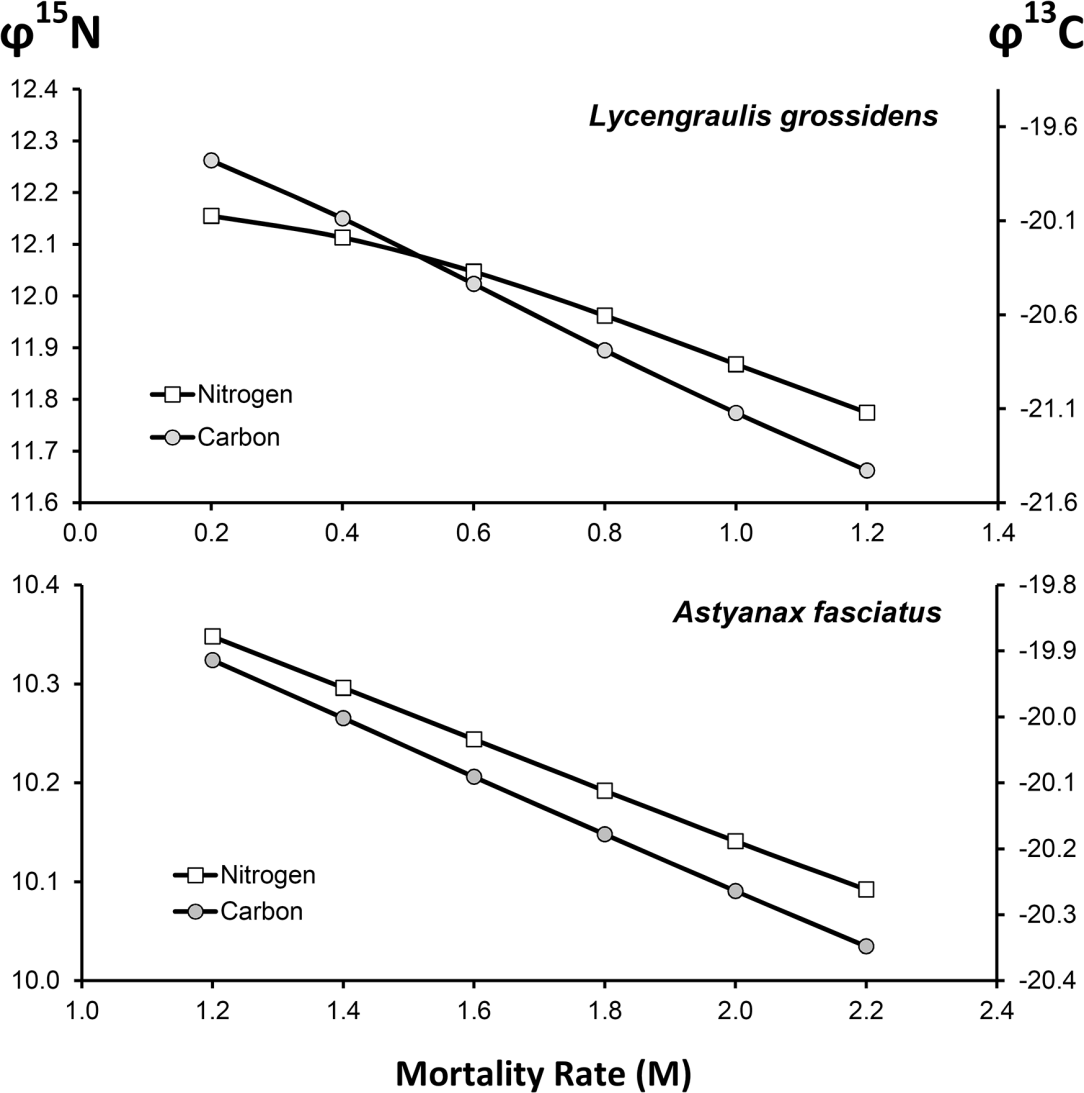
Estimates of the Weighted Isotopic Signature (*φ* ^15^ *N* or *φ* ^13^ *C*, ‰) for *Lycengraulis grossidens* and *Astyanax fasciatus* at different mortality rates, keeping the growth parameters constant.

These differences will increase depending on mortality rates. If growth parameters are kept constant, *φ* ^*15*^ *N* and *φ* ^*13*^ *C* values are closer to juvenile values if mortality rates are high, whereas these values increase more closely to the adult profile as mortality rates decrease (Fig 3).

## Discussion

Considerable effort has been expended in attempting to depict energy and matter flow in many aquatic and terrestrial ecosystems. This is the main focus in any attempt to understand ecosystem functioning and to assist in ecosystem management. The task involves the analysis of many species, from producers to top consumers, some of which are difficult to capture or rare in nature. Also, analyzing the stomach contents of only a few animals provides a very limited idea of the species’ ecological role. In this regard, the use of stable isotopes was welcomed as a time-saving methodology, because an individual’s signature could provide a picture of its long-term consumption, including the sources and mass balance [20].

Owing to budget limitations or sampling difficulties, the literature is full of examples where a species’ isotope signature is estimated from relatively small samples. Unfortunately, to depict a species’ functional role from the isotope signature is not as simple as one would desire. In addition to the type of tissue sampled and its turnover ratio [21, 22], bias can also arise from the individual preference for one or very few food items [23, 24]. Moreover, the ontogenetic shift in a species’ feeding habit and isotope signature [10, 11, 25] poses additional challenges, as the signatures of carbon and nitrogen do not vary equally: an individual can change food sources from the same trophic level (e.g., producers such as algae and macrophytes), influencing the carbon signatures; or can change the food trophic level (e.g., seeds and eggs), increasing the nitrogen variability.

*C*. *alburnus* does not show an ontogenetic shift in feeding habit: both juveniles and adults feed mainly on cladocerans, copepods and fish eggs [26]. *A*. *fasciatus* is reported to vary its main food source in relation to fish size, season and site, although it eats mainly leaves, seeds and insects [27]. *L*. *grossidens*, on the other hand, changes its feeding habits from cladocerans when small (<6 cm), adding copepods and amphipods as juveniles (6–9 cm), and restricting its food items mainly to prawns and fish when adult [18].

One side effect of this methodology is the requirement for data on growth and mortality, which are not available for most species, especially for areas with high biological diversity. Nevertheless, as data for species' size ranges are usually known, estimates of the mortality rate (*M*), asymptotic size (*L∞*) and growth constant (*K*) can be easily obtained by using the FishBase life-history tools [17]. Although these estimates are based on generalized models, the results are good enough for most species, and could be used as approximations if primary information is not available.

Another problem concerns the use of sophisticated mathematics to obtain a number that frequently is not far from a simple average from a set of data comprising animals in a wide size range. Also, by requiring a large set of parameters, the estimation of confidence intervals for each *φ* ^*15*^ *N* or *φ* ^*13*^ *C*, taking into account that the estimates depend on integrals of relatively complex functions, is a highly demanding procedure, and it is necessary to develop dedicated routines bootstrapping the available data set (there is no algebraic solution for errors). This problem of error estimates becomes even more complicated if growth and mortality parameters are obtained from FishBase, because these errors are unrealistic, expressing only the statistical errors of the generalized models, and not a specific confidence interval for each parameter.

In view of these problems, why should one use the proposed methodology? Why not simply depict a species in functional size classes, each one with a more-stable isotopic signature? The answer is related to our need to simplify nature, in order to visualize patterns, especially for highly diverse ecosystems. Usually, food webs are complex representations of ecosystem functioning, even if each species appears only once, and mainly express the feeding profile or isotopic signature of adult animals. This approach can simplify the number of elements, but also gives rise to a biased food web, where adults could be important for several aspects, such as for top-down control, but due to their low numerical importance may not represent the complete role of the species in nature.

By fractioning a species into functional groups, according to size/age classes, a better result could be achieved, but no researcher will be able to represent a food web in minimally diverse ecosystems. Also, to properly quantify the energy and matter flow in an ecosystem, the relative abundance of each functional group must be known. Of course, most of this limitation is due to our need (or habit) to represent nature in printed charts. For an n-dimensional digital model, where only small segments can be inspected each time, there is no limitation on the complexity of the food web, and partitioning a species into functional groups is highly desirable. But if growth and mortality data are available, a better result for digital models could be attained if a species is not represented by a single number, or a set of numbers, but by a mathematical function describing the species' full response to and role in the ecosystem. In this case, the species' role is better described by the species' composite impact per recruit, expressed through Eq 8.

Also, if the number of functional elements is a problem, especially if it is desired to run mixing models [20], the proposed methodology to represent a species through a simple number may be the best way to proceed. If only the simple isotopic average of a set of individuals is used, the trophic position, or the main food sources identified, will be biased both by the size profile of the sample, leading to incorrect interpretation of the species' role in the ecosystem, and by propagating errors to higher trophic levels in a food web being studied. One way to proceed, if a single number is desired, is to try unbiased samples, using a complete range of sizes and relative abundances as close as possible to the actual size distribution in nature. With this precaution, a simple isotopic average of the individuals sampled will be very close to the estimated Weighted Isotopic Signature (*φ* ^*15*^ *N* or *φ* ^*13*^ *C*), avoiding unnecessary mathematical treatment or estimates of population parameters (*M*, *L∞*, *K*, *a*, *b*), and also providing direct error estimates. In this regard, the WIS metrics (*φ* ^*15*^ *N* or *φ* ^*13*^ *C*) could be estimated as simple averages from relatively unbiased samples, or be derived from ontogenetic, growth and mortality information, as proposed in the present study.

## Supporting Information

S1 Table Stable isotope ratios of carbon and nitrogen (δ^15^N, δ^13^C) of measured specimens of *Lycengraulis grossidens* (silver sardine), *Cyanocharax alburnus* (white lambari) and *Astyanax fasciatus* (red-tail lambari) captured in Pinguela Lagoon, southern Brazil (29°49'S, 050°10'W; November and December 2009, and March and April 2010). Stable isotope ratios were quantified from dried muscle samples by the UC Davis Stable Isotope Facility, Department of Plant Sciences at the University of California.
(XLSX)
